# Supplementary material for: Bi-Layered Polymer Carriers with Surface Modification by Electrospinning for Potential Wound Care Applications
Source: Pharmaceutics. 2019 Dec 12;11(12):678. doi: 10.3390/pharmaceutics11120678 (PMC6969931; doi:10.3390/pharmaceutics11120678)
Supplement: Supplementary file 1 [file pharmaceutics-11-00678-s001.pdf]

pharmaceutics

MDPI

Supplementary Materials: Bi-Layered Polymer Carriers with Surface Modification by Electrospinning for Potential Wound Care Applications

Mirja Palo, Sophie Rönkönharju, Kairi Tiirik, Laura Viidik, Niklas Sandler and Karin Kogermann

Visualization

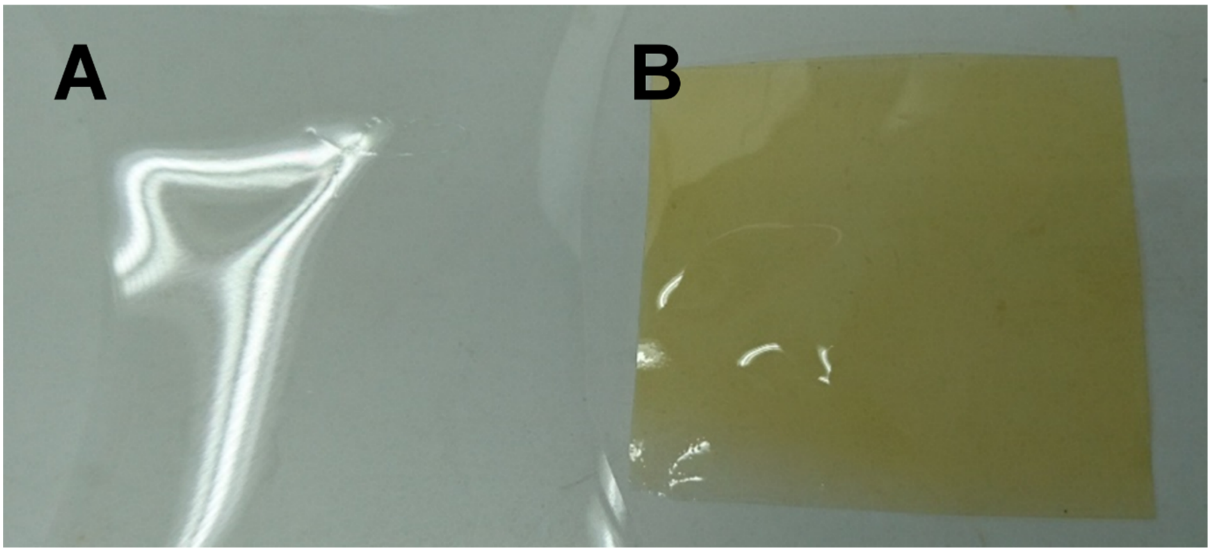

Figure S1. Photographic images of non-crosslinked (A) and crosslinked (B) solvent cast (SC) films.

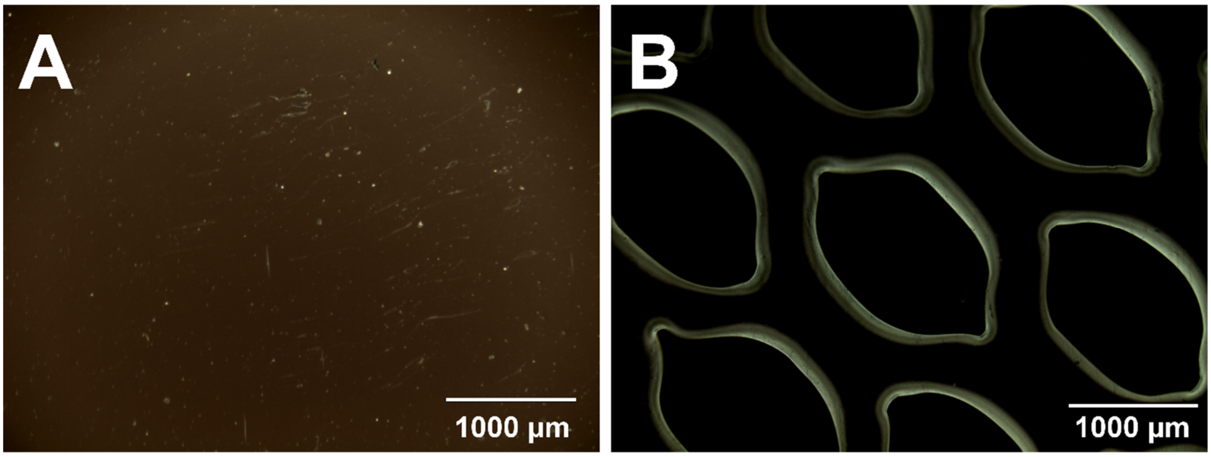

Figure S2. Optical microscopy images of non-crosslinked solvent cast (SC) film (A) and bi-layered solvent cast/3D printed (SC/3D) carrier (B).

Cell safety and viability

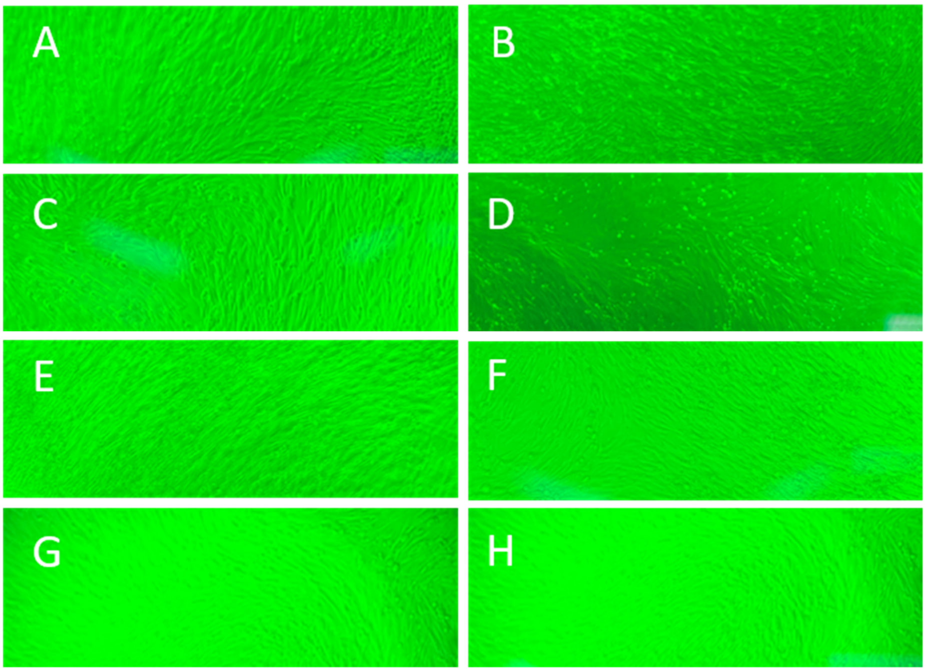

Figure S3. Optical microscopy images of baby hamster kidney (BHK-21) fibroblast cells after 24 h of incubation on top of crosslinked solvent cast (SC) film (A), bi-layered electrospun (SC/NF) carrier (C), 3D-printed (SC/3D) carrier (E) and non-crosslinked SC film (B), bi-layered electrospun (SC/NF) carrier (D), 3D-printed (SC/3D) carrier (F) together with positive healthy controls: pure medium with a glass-plate (G) and pure medium in plastic well (H).
